# Ibudilast sensitizes glioblastoma to temozolomide by targeting Macrophage Migration Inhibitory Factor (MIF)

**DOI:** 10.1038/s41598-019-39427-4

**Published:** 2019-02-27

**Authors:** Wendy Ha, Hatice Sevim-Nalkiran, Ashraf M. Zaman, Kazuko Matsuda, Mustafa Khasraw, Anna K. Nowak, Liping Chung, Robert C. Baxter, Kerrie L. McDonald

**Affiliations:** 1 Cure Brain Cancer Foundation Biomarkers and Translational Research Group, Prince of Wales Clinical School, Lowy Cancer Research Centre, University of New South Wales, Sydney, NSW Australia; 2 Department of Medical Biology, Faculty of Medicine, Recep Tayyip Erdogan University, Rize, Turkey; 3 MediciNova Inc, La Jolla, CA USA; 4 Royal North Shore Hospital, Department of Medical Oncology, University of Sydney, St Leonards, NSW Australia; 5 School of Medicine, University of Western Australia, Crawley, WA Australia; 6 Kolling Institute of Medical Research, University of Sydney, St Leonards, NSW Australia

## Abstract

Recurrence in patients with glioblastoma (GBM) is inevitable resulting in short survival times, even in patients with *O-6-Methylguanine-DNA Methyltransferase* (*MGMT*) methylation. Other pathways must be activated to escape from temozolomide (TMZ) treatment, however acquired resistance mechanisms to TMZ are not well understood. Herein, frozen tumors from 36 *MGMT* methylated patients grouped according to overall survival were extracted and proteins were profiled using surface-enhanced laser desorption/ionization (SELDI) with time-of flight (TOF) proteomics to identify low molecular weight proteins that associated with poor survival outcomes. Overexpression of macrophage migration inhibitory factor (MIF) was identified in human GBM specimens that were *MGMT* methylated but showed poor survival. This correlation was confirmed in an independent cohort of human GBM. MIF overexpression has been reported in several cancer types, including GBM. We repurposed ibudilast, a specific MIF inhibitor, and treated patient derived cell lines. Ibudilast showed modest anti-proliferative activity however, when combined with TMZ, significant synergism was observed, resulting in cell cycle arrest and apoptosis. *In vivo*, combined ibudilast and TMZ treatment of a patient derived xenograft (PDX) model resulted in significantly longer overall survival. Our findings have significant clinical implications for people with GBM. Since clinical trials involving ibudilast have shown no adverse side effects and the drug readily penetrates the blood brain barrier, treatment of GBM with this combination is clinically achievable.

## Introduction

Brain cancers affect people of all ages and are the most common cause of cancer death in children and young adults^1^. People with grade IV glioma (glioblastoma or GBM) face a poor prognosis and inevitable tumor recurrence. GBM has a median survival of less than 15 months^2^. The current optimal treatment for people with GBM who are fit consists of maximal safe surgical resection (gross total resection; GTR) then external beam irradiation with concomitant temozolomide (TMZ). This is followed by 6 months of TMZ therapy, which results in a 2.5-month improvement in survival over radiotherapy alone^2^.

Methylguanine-DNA methyltransferase (MGMT) removes mutagenic alkyl adducts, thereby protecting DNA from the damage induced by TMZ and other alkylating agents. Loss of MGMT expression is a frequent event in various human cancers^3^. MGMT expression in cells is regulated by hypermethylation of the CpG islands within the promoter and enhancer regions of the gene^4–6^. In tumors with a methylated *MGMT* promoter, MGMT deficiency is presumed, resulting in the enhanced effects of TMZ^7^.

Detection of *MGMT* promoter methylation correlates strongly with clinical response to TMZ and is also a positive prognostic biomarker in TMZ-treated GBM, including in elderly patients^8,9^. There has been considerable enthusiasm to use MGMT as a predictive biomarker for GBM patients, with the long-term scope for its use as a biomarker to assign alkylating therapy to individual patients, and it is an important stratification factor in current clinical trials. However, even patients with *MGMT* promoter methylated tumors eventually progress and succumb to their disease^10^. Given progression occurs in *MGMT* promoter methylated tumors^4^, this indicates that other pathways must be activated to escape from TMZ treatment, however acquired resistance mechanisms to TMZ are not well understood. The tumor suppressor p53 (p53)^11^, mismatch repair (MMR) deficiencies^12^ and microRNA (miRNA)^13^ are all well studied mechanisms of resistance. However, despite a plethora of pre-clinical studies, resistance to TMZ has not been clinically addressed.

We took an unbiased approach by selecting human GBM tumors that were *MGMT* methylated, responded initially to TMZ treatment, and grouped them according to patient survival. We used surface-enhanced laser desorption/ionization (SELDI) with time-of flight (TOF) proteomics to identify low molecular weight proteins that associated with poor survival outcomes. Macrophage Migration Inhibitory Factor (MIF) was strongly expressed in tumors from those patients with shorter survival despite *MGMT* methylation. By targeting MIF with a specific inhibitor, we show herein, that we can sensitize tumors to TMZ treatment in patient derived cell lines and a patient derived xenograft model.

## Results

### Identification and validation of MIF expression as a marker of poor prognosis

**Figure 1:**
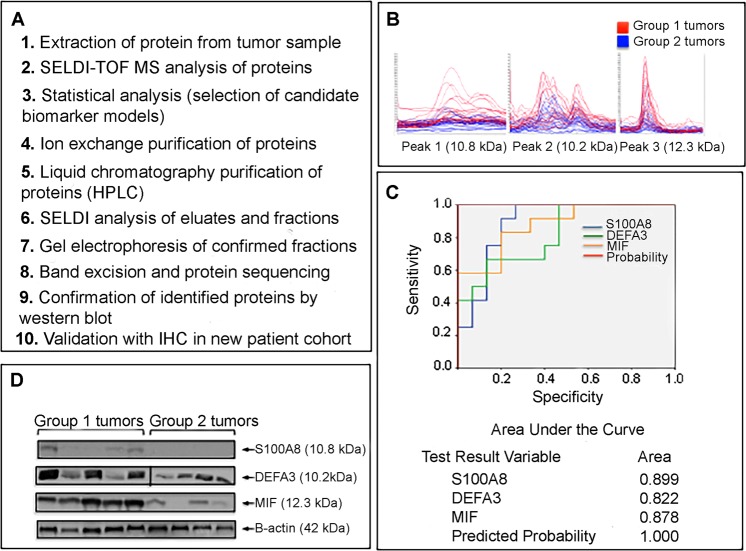
Identification of Macrophage Migration Inhibitory Factor (MIF) in GBM patient samples. (**A**) Flow chart of the steps taken to identify MIF using protein lysates extracted from GBM *MGMT* methylated patient specimens (n = 36). (**B**) SELDI-TOF MS spectra demonstrating 3 differentially expressed protein peaks in Group 1 tumors (survival <13.8 months; n = 18) and Group 2 tumors (survival >13.8 months; n = 18). Peaks in red belong to proteins overexpressed in Group 1 tumors. (**C**) Receiver Operating Characteristic (ROC) analysis for the 3 proteins (S100A8 [blue], DEFA3 [green] and MIF [yellow]) to discriminate Group 1 tumors from Group 2 tumors. The Area Under the Curve (AUC) values are provided. **(D)** Representative western blots depicting changes in protein expression in lysates from Group 1 (n = 5) and Group 2 (n = 4) tumors. Untreated lysates were extracted from frozen GBM specimens and probed with the indicated antibodies.

We selected 36 frozen newly diagnosed GBM specimens that were *MGMT* promoter methylated. Our reasoning behind selecting only methylated patients was that we wanted to see an initial response to TMZ. All patients had undergone maximal safe resection and were treated with concurrent RT and TMZ followed by adjuvant TMZ (Table 1). Sixteen patients were treated with salvage chemotherapies at progression including carboplatin, lomustine and bevacizumab. The median survival of the cohort selected was 13.8 months. The cohort was further categorized into two groups based on the median survival; Group 1: survival <13.8 months (n = 18) and Group 2: survival ≥13.8 months (n = 18). Proteins were extracted and subjected to SELDI-TOF MS (Fig. 1A).

**Table 1:** Clinical features of GBM patients enrolled in the proteomics analysis.

	Group 1 (survival <13.8 months)	Group 2 (survival >13.8 months)	p-value
	n = 18	n = 18	
**Gender**			
Male (%)	90	80	
Female (%)	10	20	
**Age**			
Median (years)	66.9	61.4	0.025
Range (years)	50–75	31–83	
XRT/TMZ (%)	100	100	
Treatment given at progression (%)	33	55	0.0002
MGMT methylation (%)	100	100	
Survival			
Median (months)	10.53	20.83	0.0023
Range (months)	2.5–13.4	13.9–50.3	

Abbreviations: XRT/TMZ: Concurrent radiotherapy and temozolomide (TMZ) plus adjuvant temozolomide.

We found 39 peaks to be differentially expressed between the groups, with 7 identified peaks down-regulated and 32 peaks up-regulated in Group 1 (Fig. 1B). Peak clusters with high p-value, AUC and peak quality were subjected to backward stepwise BLR analysis in order to generate a reliable biomarker panel and to evaluate the combined discrimination power of the selected biomarker candidates. A panel of significantly up-regulated proteins in Group 1 (m/z 10247, 12361 and 10850 Da) was found to be the best model capable of distinguishing Group 1 from Group 2 tumors with 100% sensitivity and specificity (Fig. 1C). These three proteins were further purified for identification using ion-exchange separation and peptide matches were detected using liquid chromatography tandem mass spectrometry (LC-MS/MS). Mascot search reporting of the protein tryptic digests identified specific sequences of the 3 selected biomarkers (10.2, 12.3 and 10.8 kDa) to be alpha Defensin-3 (DEFA3), macrophage migration inhibitory factor (MIF) and calgranulin A (S100A8). Primary antibodies to the proteins confirmed the identity and overexpression in protein lysates from Group 1 and Group 2 patients (Fig. 1D). From the Western blot analysis, MIF expression was clearly distinguishable between Group 1 and Group 2 tumors and thus the remainder of our studies focused on MIF. ROC curve analysis was performed for MIF as an individual biomarker. The predictive value of MIF was 0.882 with an asymptotic significance of >0.01, indicating good discriminating ability.

**Figure 2:**
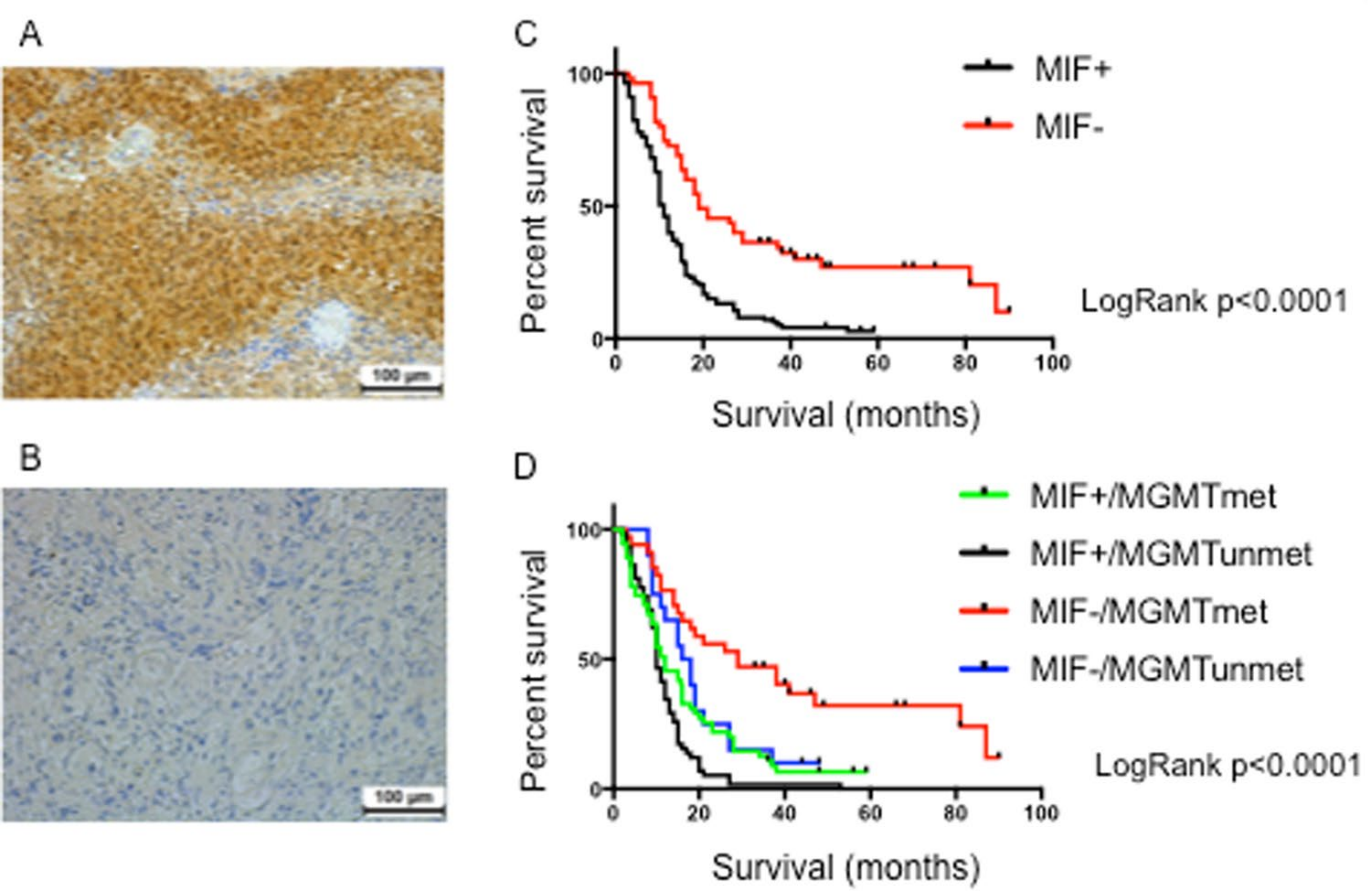
Evaluation of MIF protein expression in an independent cohort of GBM. (**A**) Photomicrographs are representative of positive MIF staining and (**B**) negative MIF staining. Photos were taken at a magnification of x20 and reviewed blindly by 2 independent pathologists. Bar represents 100 μm. Approximately 111 out of 168 GBM (66%) stained positive for MIF. (**C**) Significant differences in the overall survival times (months) for patients with MIF + (n = 111) and MIF- (n = 57) GBM tumors as depicted by the Kaplan Meier survival curves. LogRank: p < 0.001. (**D**) Patients were stratified into 4 groups based upon *MGMT* promoter methylation status: (Group 1: MIF+/*MGMT* methylated n = 55; Group 2: MIF+/*MGMT* unmethylated (n = 58); Group 3: MIF−/*MGMT* methylated (n = 24); Group 4: MIF−/*MGMT* unmethylated (n = 18). Significant survival benefit can be seen when MIF negative tumors are grouped by *MGMT* status. LogRank: p < 0.0001.

We assessed the protein expression of MIF in an independent cohort of 168 human GBM samples using IHC. The clinical features of patients from whom these samples were derived are summarized in Table 2. MIF staining was specific to the tumor cell membrane, as well as cytoplasm (Fig. 2A,B). One hundred and thirteen (67%) patients stained positive for MIF and was significantly associated with poor survival outcomes (Fig. 2C). MIF expression has been previously associated with poor prognosis and early tumor recurrence in GBM^14–16^. Patients whose tumors were negative for MIF showed a median survival time of 19 months compared to 11 months for MIF positive tumors (LogRank p < 0.0001). The hazard ratio was 2.4 (1.74–3.31) for patients with MIF positive tumor tissue. For all 168 GBM specimens, *MGMT* promoter methylation status was known. The patients were categorized into 4 groups: MIF + ve and *MGMT* methylated (n = 55); MIF + ve and *MGMT* unmethylated (n = 58); MIF-ve and *MGMT* methylated (n = 24); MIF-ve and *MGMT* unmethylated (n = 17). *MGMT* methylation status was irrelevant in the MIF + ve tumors (median survival 12 months versus 10 months for *MGMT* methylated and unmethylated tumors) (Fig. 2D). However, methylation of the *MGMT* promoter was strongly associated with better survival in the group of MIF-ve tumors (29 months versus 17 months) for *MGMT* methylated and unmethylated tumors, respectively. This confirms our pilot study (Fig. 1) where we showed MIF + ve/*MGMT* methylated tumors were associated with poorer survival (Group 1 tumors).

**Table 2:** Clinical Features of GBM patients enrolled in the immunohistochemistry analysis.

	MIF−	MIF +	p-value
	n = 57	n = 111	0.0002
**Gender**			
Male (%)	49	66	
Female (%)	51	3	
**Age**			
Median (years)	56	59.5	0.059
Range (years)	25–81	33–85	
**MGMT**			
Methylated (%)	54	50	0.010
Unmethylated (%)	46	50	
XRT/TMZ (%)	100	100	
**Survival**			
Median (months)	19.0	11.0	<0.0001
Range (months)	(2.7–49.2)	(2.2–81.3)	

Abbreviations: XRT/TMZ: Concurrent radiotherapy and temozolomide (TMZ) plus adjuvant temozolomide.

### Ibudilast treatment sensitizes GBM cells to Temozolomide

To expedite rapid translation to the clinic, we performed a literature search to identify known pharmacological inhibitors of MIF. Ibudilast (MN166) is an orally available, small molecule drug and is a selective inhibitor of MIF^17^. Ibudilast distributes well to the CNS^18^ and inhibits MIF at clinically-relevant plasma or CNS concentrations. A panel of 8 patient derived cell lines (*MGMT* methylated: BAH1, G53, HW1 or *MGMT* unmethylated: RN1, G57, G89, G244) were tested with increasing concentrations of ibudilast (0–800 μM) and cell proliferation was measured with the MTS assay after 72-hours. The IC_50_ values ranged from 40–120 μM (data not shown).

**Figure 3:**
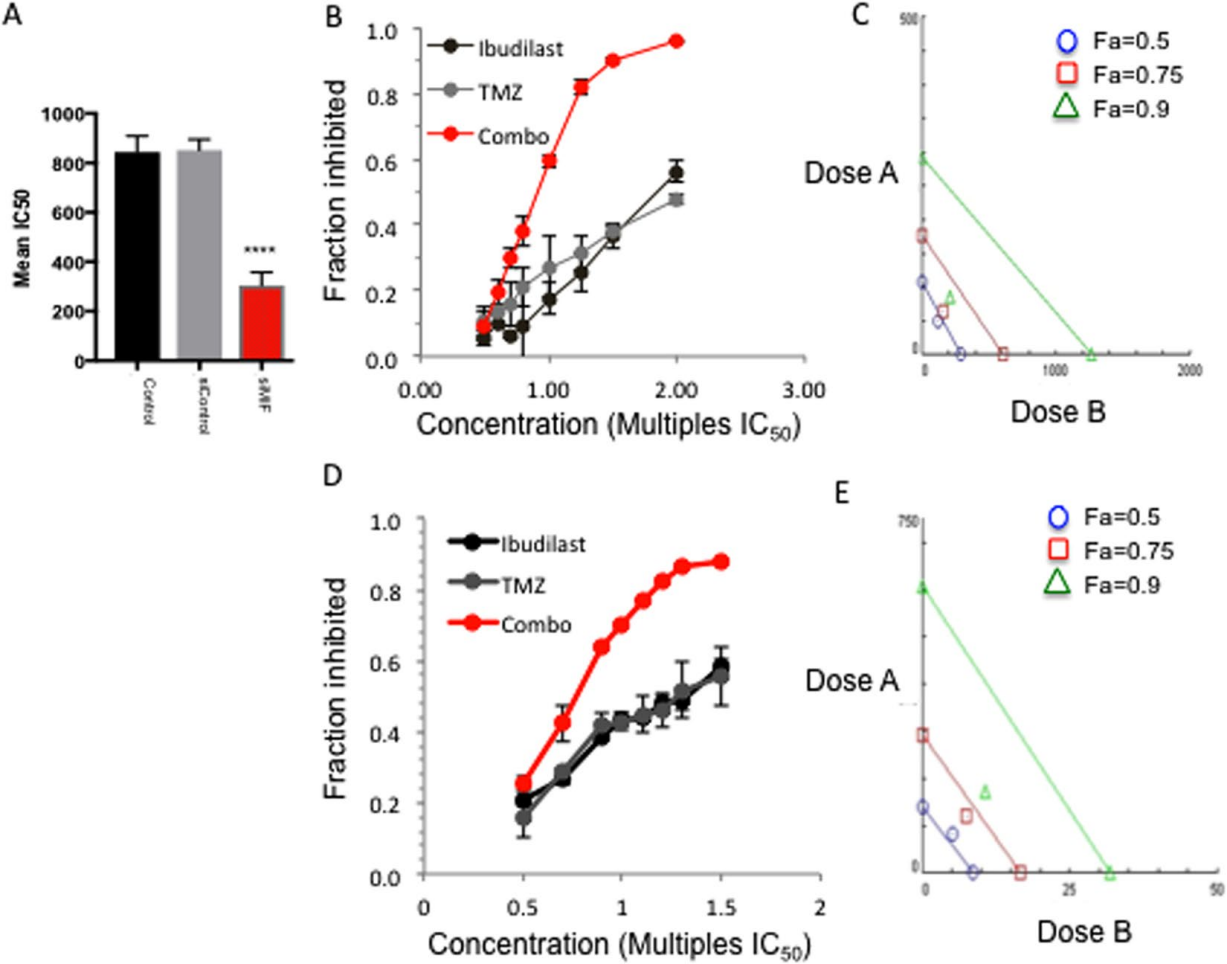
Combined TMZ and MIF inhibitor (ibudilast) treatment is synergistic in GBM cell lines. Different fractions of the monotherapy IC_50_ doses of ibudilast and TMZ were combined (red line). Cells were treated for 7 days and the cell fraction inhibited was determined with the CellTitre 96® Aqueous Assay. (**A**) RN1 *MGMT* unmethylated patient derived cells and (**B**) BAH1 *MGMT* methylated patient derived cells. (**C**,**D**) Isobolograms were generated using CompuSyn 1.0 software. Using this method, the dose–effect data of the individual drugs measured above were used to determine the expected combination and then statistically compared to the actual combination effect measured to determine whether there was synergism, additivity, or anti-additive interactions. These results are expressed in an isobologram that graphs the effective doses of inhibition at 50% (*F*_a_ 0.5), 75% (*F*_a_ 0.75), and 90% (*F*_a_ 0.9) for the individual drugs as *x*- and *y*-intercept values. Synergism is demonstrated in all cell lines by the dose pair plotting as a point (*symbol*) below their respective *F*_a_ isobole or line. Data depicted are the mean ± SEM from 3 replicate experiments.

Combining ibudilast with TMZ *in vitro*, proliferation was significantly inhibited in all cell lines compared to TMZ treatment alone (RN1 and BAH1 cells are shown in Fig. 3). Isobologram analysis using CompuSyn software determined that the enhanced effect of the combined treatment was synergistic for at least one of the fractional effect (Fa) doses demonstrated in the isobolograms and determined by the CI values (Table 3) for both cell lines.

**Table 3:** Combination Index (CI) values for ibudilast/TMZ combination treatment.

Cell line	CI Values		
	Fa = 0.50	Fa = 0.75	Fa = 0.90
RN1	0.879	0.631	0.460
BAH1	1.184	0.857	0.500

Synergistic (CI < 1), Additive (CI = 1), Antagonsistic (CI > 1).

**Figure 4:**
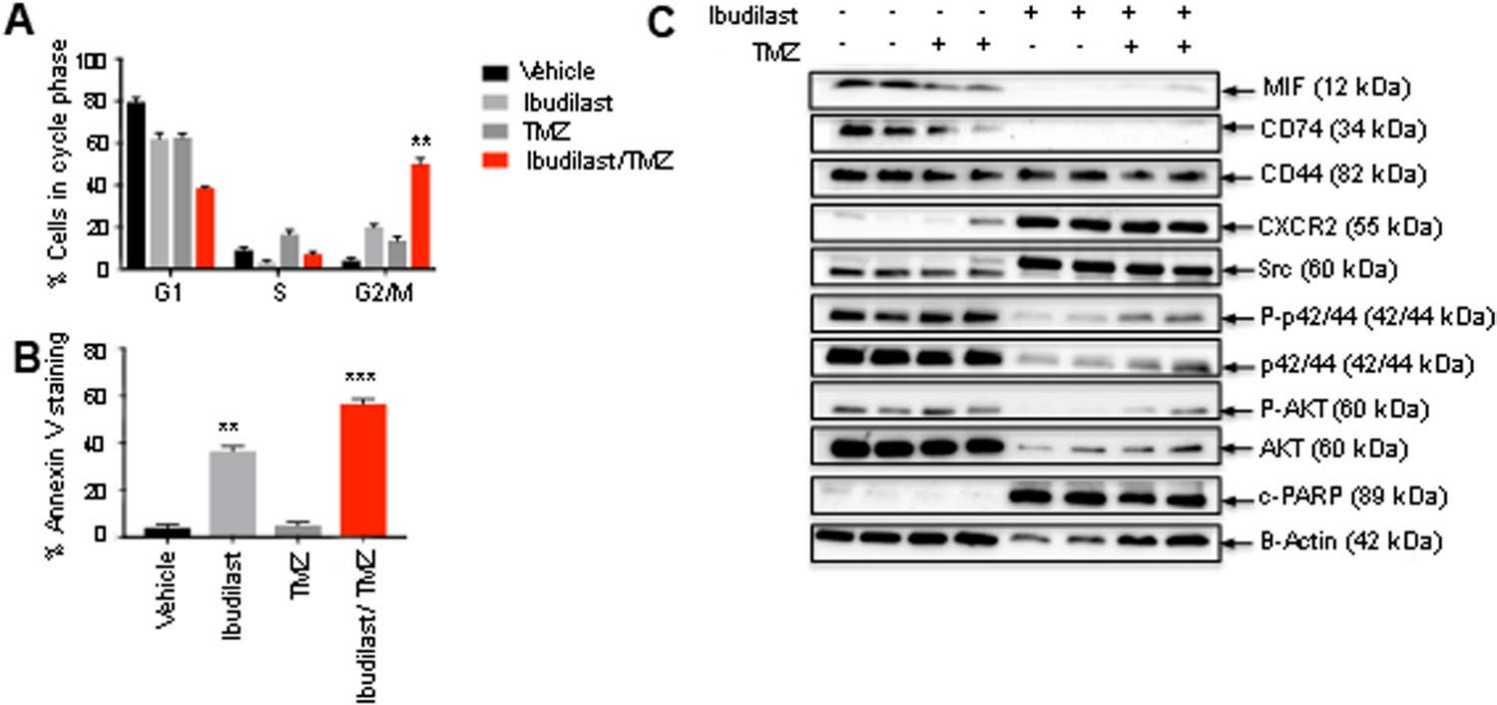
Combined ibudilast and TMZ treatment results in cell cycle arrest and induces apoptosis in GBM cell lines. RN1 cells were treated *in vitro* for 7-days with ibudilast (195 μM), TMZ (110 μM) or combined treatments. (**A**) Cell cycle analysis in GBM cell lines treated with vehicle, ibudilast, TMZ or the combination. Data depicted are the mean ± SEM. *p < 0.05, **p < 0.01, ***p < 0.001, ****p < 0.0001 by one-way ANOVA comparing the cells in G_0_/G_1_ between treatment groups. Three replicate experiments were conducted. (**B**) RN1 cell lines were treated with vehicle, ibudilast, TMZ or the combination for 7-days and analyzed using flow cytometry for Annexin V positive, propidium iodide (PI) negative cells. Data depicted are the mean ± SEM. *p < 0.05, **p < 0.01, ***p < 0.001, ****p < 0.0001 by one-way ANOVA. Three replicate experiments were conducted. (**C**) Representative western blots depicting changes in protein expression in RN1 cells following 7-day treatment with vehicle, ibudilast, TMZ or the combination. Lysates were made and probed with the indicated antibodies.

We next examined combined ibudilast and TMZ treatment on cell cycle and apoptosis *in vitro* using the *MGMT* unmethylated cell line, RN1. Combined treatment significantly increased the effect on G_1_ cell cycle arrest compared with single agent ibudilast and vehicle treatments (Fig. 4A). Notably, combined treatment with ibudilast and TMZ significantly increased apoptosis as indicated by an increase in percentage of Annexin V^+^/propidium iodide^−^ cells compared with single-agent and vehicle treatments (Fig. 4B). Western blot analysis confirmed the specificity of ibudilast on MIF, with expression significantly inhibited in the presence of the drug compared to vehicle-treated control cells (Fig. 4C; Supplementary Fig. 1). MIFs target receptor, CD74 was also inhibited in the presence of ibudilast. Combining ibudilast with TMZ reduced the levels of total and phosphorylated AKT and ERK (p44/42). Src physically associates with the intra-cytoplasmic domain of CD44, leading to phosphorylation, which then triggers downstream RAS/RAF/MEK/MAPK signaling. Increased Src expression was observed in the cells treated with the combination treatment. MIF-CD74 can complex with cysteine-X-cysteine (CXC) chemokine receptor, CXCR2. As can be seen in Fig. 4C, expression levels of CXCR2 were increased when exposed to ibudilast in combination with TMZ. Increased cleaved PARP, an indicator of apoptosis, increased with ibudilast and the combination treatment, consistent with that observed in Fig. 4B.

### Ibudilast combined with Temozolomide improves survival times *in vivo*

**Figure 5:**
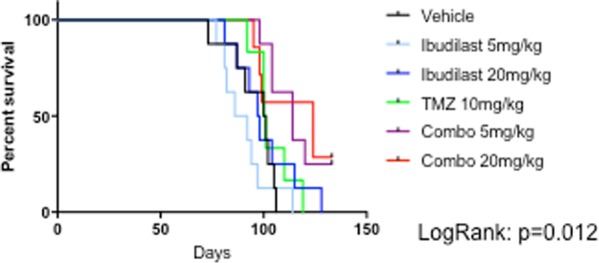
MIF inhibition in combination with ibudilast and TMZ treatment results in longer survival *in vivo*. Survival of RN1 PDX model. Tumor-bearing mice were treated with indicated drug combinations with the following doses: Ibudilast (5 mg/kg); Ibudilast (20 mg/kg); TMZ (10 mg/kg); Ibudilast (5 mg/kg) + TMZ (10 mg/kg) and Ibudilast (20 mg/kg) + TMZ (10 mg/kg). There were n = 8 mice in all groups. All treatments ceased by day 100. Survival data is displayed by the Kaplan-Meier curve. LogRank: p = 0.012. Tumors were harvested from all mice in the treatment groups and fixed in formalin for paraffin embedding and sectioning.

To examine whether combined treatment impacted on survival of tumor-bearing animals *in vivo*, we implanted viable RN1 cells intracranially into immunocompromised Balb/c mice. Two doses of ibudilast were chosen: 5 mg/kg and 20 mg/kg delivered by oral gavage while TMZ was administered by i.p. injection (10 mg/kg)^19^. Tumor was confirmed by humanely euthanizing mice at different time-points during the experiment and checking for tumor with H&E staining. At 43 days post-implantation, large tumors were present and treatment commenced. When all vehicle-treated mice reached their neurological endpoint (median survival 100.5 days), treatment was stopped. The treatment of tumor-bearing mice with ibudilast only resulted in inferior median survival times compared to the vehicle-treated mice (89 days and 97.5 days respectively) (Fig. 5A). A survival advantage was observed with mice treated with TMZ alone (median survival: 105.5 days compared to 100.5 days; LogRank p = 0.055). Combined treatment resulted in significantly longer survival. Mice treated concurrently with ibudilast (5 mg/kg) and TMZ (10 mg/kg) displayed a median survival of 114 days (p = 0.005) while the combination of ibudilast (20 mg/kg) and TMZ (10 mg/kg) resulted in a median survival of 111.5 days (p = 0.014).

**Figure 6:**
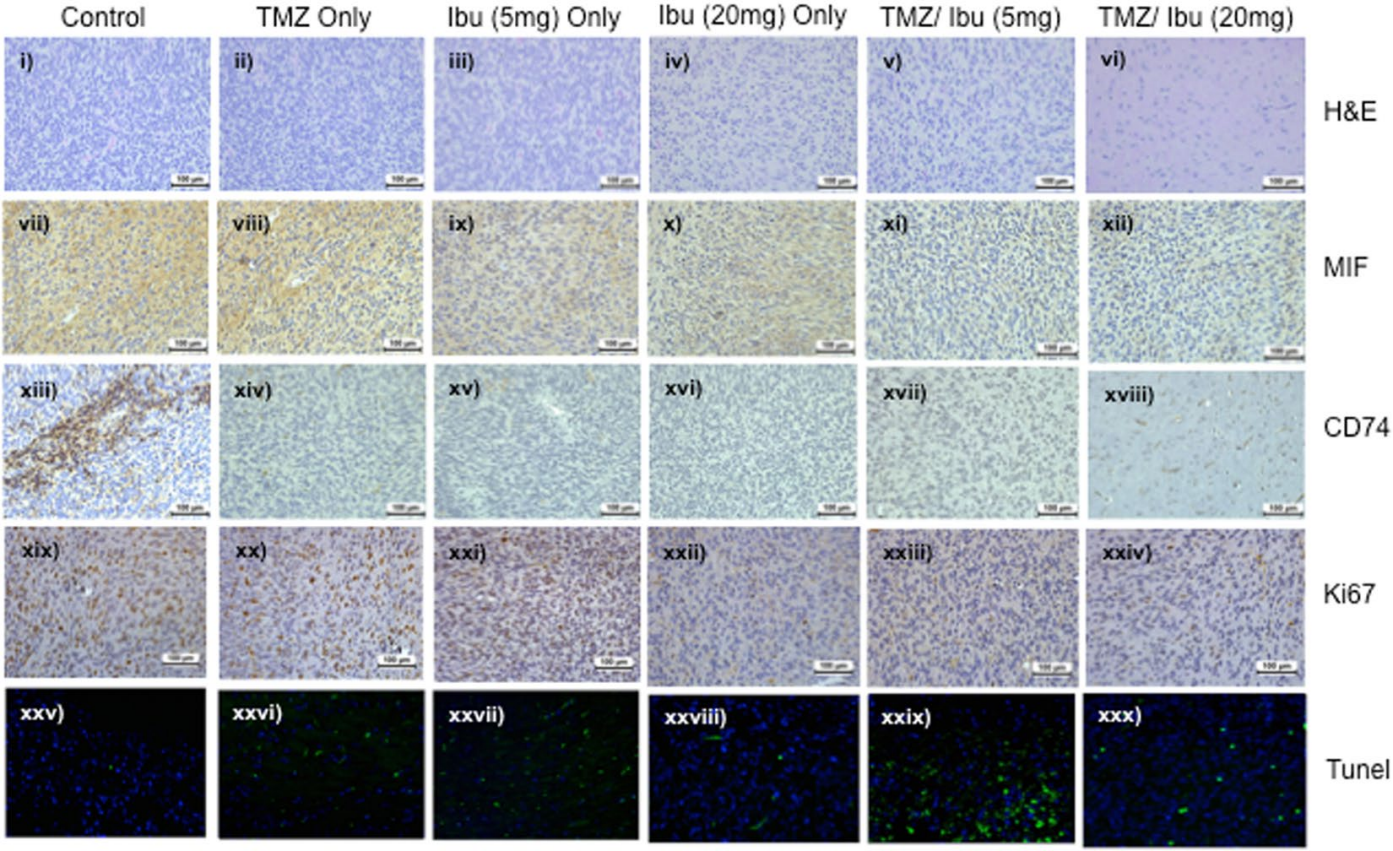
Immunohistochemistry and immunofluorescence analysis of excised mouse brain derived from the *in vivo* experiment. Brains from the treated mice (n = 8 per treatment group) were harvested at the time of euthanasia. Sections (4 μm) were cut and mounted on ultrafrost slides. H&E, MIF, CD74 and Ki67 proteins were assessed with IHC while Tunel positivity was assessed with immunofluorescence (IF). Representative photomicrographs are presented from each treatment group (i–iv H&E; vii–xii MIF; xiii–xviii CD74; xix–xxiv Ki67 and xxv–xxx Tunel). All images were taken at a magnification of x20 and the error bars represent 100 μm.

All brains were harvested from the mice and processed for IHC and immunofluorescence (Tunel). Figure 6 illustrates representative photomicrographs of the harvested brains after staining for: H&E, MIF, CD74, Ki67 and Tunel. Histologically, the brains harvested from the combination treatments showed less mitotic bodies compared to the controls and other treatment groups. Marked reductions in the expression of MIF and CD74 were observed in all treatment arms (with the exception of TMZ only) compared to the control brains. MIF and CD74 expression was not detected in any of the mice brains harvested from the combination arms. The proliferation index marker, Ki67 was used to determine tumor activity. Ki67 is a nuclear stain and was detected in approximately 10–20% of the brain sections harvested from control mice, TMZ only treated mice and the two ibudilast only treatments. In contrast, Ki67 was detectable in less than 5% of tumor cells in the brains excised from combination-treated mice. Tunel staining of the vehicle-treated mice tumors revealed very low levels of apoptosis (<5%). Concurrent treatment of mice with ibudilast (both concentrations) and TMZ resulted in a 30% increase in Tunel positive cells, indicating active apoptosis in the treated mice tumors.

## Discussion

Our findings have significant clinical implications for people with GBM. First, we found patients who relapsed after TMZ treatment had high levels of MIF expression. Secondly, ibudilast, a MIF-specific inhibitor sensitized cells and tumors to TMZ treatment. Since clinical trials involving ibudilast have shown no adverse side effects and the drug readily penetrates the blood brain barrier, treatment of people with glioma with this combination is clinically achievable.

MIF expression has been previously associated with poor prognosis and early tumor recurrence in GBM^14–16^. The evaluation of a series of bioinformatics databases demonstrated significantly elevated levels of MIF in GBM compared with nontumor tissue and high levels of MIF correlated with poorer survival^20^. Binding of MIF to the extracellular domain of the non-polymorphic type II integral membrane protein CD74 is necessary as an initial step for the MIF signaling cascade^21^. There are two major distinct MIF-CD74 interactions; (1) Binding of MIF to CD74 can induce the recruitment of CD44 to the complex, which then activates non-receptor tyrosine kinases, leading ultimately to extracellular signal-regulated kinase phosphorylation and enhanced tumor growth in an autocrine manner or (2) MIF-CD74 complexes with the cysteine-X-cysteine (CXC) chemokine receptors CXCR2 on myeloid cells and CXCR4 on T cells thus influencing the M1/M2 polarization in intra-tumoral immune cells.

MIF exerts multimodal functions in GBM including pro-proliferative, pro-migratory, pro-angiogenic as well as immune-evasive properties^14,15,22^. In two independent studies, brain tumor initiating cells (BTIC) or glioma stem cells (GSC) were shown to express high levels of MIF^20,23^. In addition, heightened CD74 expression in GBM cell lines has been implicated in mediating resistance against TMZ in GBM^24^. Using short hairpin RNA to inhibit CD74 in U87 cells, sensitivity to TMZ treatment was increased^24^.

Ibudilast (MN166) is an anti-inflammatory drug and an allosteric inhibitor of MIF^17^ and is approved for clinical use in bronchial asthma and cerebrovascular disorders in Japan. We found that ibudilast alone modestly inhibited cell proliferation in a panel of patient-derived cell lines, however acted synergistically when combined with TMZ resulting in high levels of apoptosis. Based on our analysis of protein lysates collected post-treatment, ibudilast and TMZ not only blocked the interaction between MIF and CD74 but also appeared to degrade the expression levels of these proteins.

Numerous studies have shown that impeding MIF expression levels with antibodies or short interfering RNAs lead to significantly reduced tumor formation *in vivo*^14,16,20^. We waited until the tumor was grown to a substantial size before commencing treatment in contrast to investigations using siRNA or shRNA to block MIF expression^20^. The combination of ibudilast with TMZ resulted in significantly extended survival times. We reported no adverse effects from the combination treatment.

The precise mechanism(s) of how ibudilast works synergistically with TMZ to reduce tumor size and extend survival in mice is not fully clear. MIF inhibition has also been shown to sensitize osteosarcoma cells to cisplatin and doxorubicin^25^. High levels of MIF have been shown to be involved in immune evasion^15,16,26,27^. MIF transmits signals to the brain parenchyma and in particular to microglial cells^28^. Interruption of this glioma-microglial interaction through an antibody-neutralizing approach or small interfering RNA (siRNA)-mediated inhibition of MIF prolonged the survival time in glioma-implanted mice by reinstating the microglial pro-inflammatory M1 function^28^. MIF has also been suggested to act indirectly as a promoter of GBM progression, via suppression of immunological rejection by activating and protecting the negative regulators, myeloid-derived suppressor cells (MDSCs), within the tumor microenvironment^20^. In another cancer model, treatment of rhabdomyosarcoma cells with cytotoxic drugs actually increased secretory levels of MIF, leading to immune escape^27^. Hence, targeting MIF may contribute to restoration of immune sensitivity^27^. Moreover, our findings hold important clinical application. An early phase clinical trial of ibudilast in combination with TMZ for patients with recurrent GBM is underway.

## Materials and Methods

### Ethics statement

Human specimens and clinical data were collected with approval of the relevant Human Research Ethics Committee (HREC) and all patients provided written informed consent. All animal experiments were approved by the University of New South Wales Animal Care and Ethics Committee (ACEC). All methods were performed in accordance with the relevant guidelines and regulations.

### Clinical Specimens and Patient-Derived Cell lines

Fresh frozen tumor specimens (n = 36) and formalin-fixed paraffin embedded (FFPE) tumor specimens (n = 168) of human GBM were obtained from the Kolling Institute Tumour Bank and the Australian Genomics and Clinical Outcomes of Glioma (AGOG) biobanks. Clinical data is summarized in Tables 1 and 2. The patient-derived GBM cell lines, RN1 and BAH1 were provided by Dr Bryan Day (QMIR Berghofer)^29^ and grown in neurobasal serum-free media as described previously^30^. All patient-derived cell lines used in this study were authenticated by short tandem repeat (STR) analysis. Cell lines were used within 10 passages and have been verified to be negative for mycoplasma contamination. All clinical specimens and cell lines were tested for *MGMT* methylation using pyrosequencing as described previously^31^.

### Preparation of protein sample and protein chips for SELDI-TOF MS

Proteins extracted from fresh frozen GBM surgical specimens (n = 36) were analyzed by TOF MS on SELDI protein chip arrays (Bio-Rad) as previously described^32^. All mass spectra were obtained in the m/z range of 2,500–70,000 with the ProteinChip SELDI system Enterprise Edition (Bio-Rad). Mean values from duplicate spectra for each sample were used in all subsequent analyses. The m/z for each peak was determined using external calibration with protein standards as previously described^32^. After pre-processing of data, intensities of each peak in the group were averaged for each sample. Differences between samples and the two groups were determined by Expression Difference Mapping (EDM) using Data manager Software 4 (Enterprise Edition Bio-Rad). For each significantly differentially expressed protein, receiver operating characteristic (ROC) curves were constructed to obtain area under curve (AUC) values (SPSS Software Version 18.0). AUC values which range from 0.5 (no discrimination) to 1.0 (absolute prediction) were compared using the Hanley & McNeil method to evaluate discriminatory power of proteins and the significance of ROC curves^33,34^. Backward stepwise binary logistic regression (BLR) procedure was applied to compute contribution of individual markers to the separation of the two test groups and also to estimate the complimentary performance of multiple biomarkers. Protein extracts were further purified using the Mustang S cation-exchange AcroPrep 96 Filter Plate (Pall Corporation) and fractionated by Reverse Phase High Performance Liquid Chromatography (RP-HPLC) (Model 510, Waters Corporation) to obtain purified subfractions containing proteins of interest. For protein identification, samples were sent to the Bio-analytical Mass Spectrometry Facility (BMSF), University of New South Wales, Sydney, Australia.

### Immunohistochemistry (IHC) and Scoring

Immunohistochemistry (IHC) was performed with a well-characterized MIF antibody on formalin-fixed paraffin-embedded GBM specimens from 168 patients who had undergone craniotomy. Sections (4 μM) were deparaffinised and dehydrated. Human breast cancer tissue was used as a positive control. Sections were treated with peroxide block reagent (Bond polymer refine detection kit, Leica) to block endogenous peroxide activity. The sections were immunostained with MIF (MAB289, R&D Systems; 1:8000) on a Bond X automated immunostainer (Leica) for 30 minutes. Immunostaining was developed with DAB-chromagen for 10 minutes and counterstained with haematoxylin to allow nuclei visualization. Stained sections were scored by two independent pathologists. Cytoplasmic staining was scored as intensity with 0 = no staining; 1 = weak staining; 2 = moderate staining and 3 = strong staining.

### Drug compounds

MN-166 (ibudilast) was kindly supplied by MediciNova Inc. Temozolomide (TMZ) was purchased from Sigma Aldrich. All drugs for *in vitro* and *in vivo* use were reconstituted in DMSO at 10 mM. Doses used are detailed in Figure legends.

### Combination treatment synergy quantitation

Drug combinations studies were performed according to the Chou-Talalay method of synergy quantitation^35^. Patient derived cell lines were treated *in vitro* for 7-days with the combination of ibudilast and TMZ over a range of concentrations held at a fixed ratio based on the IC_50_ (drug concentration required for 50% growth inhibition) of each drug specific for each cell line. CompuSyn 1.0 performs multiple drug dose-effect calculations using the Median Effects methods described by Chou and Talalay^36^ and was used to determine the combination index (CI) which offers quantitative definition for additive effect (CI = 1), synergism (CI < 1), and antagonism (CI > 1) of drug combinations.

### Flow cytometry analysis

Cells were plated in 24-well plates and treated the following day with the indicated agents. After treatment with drugs, cells were harvested at 7-days and fixed in cold 70% v/v ethanol overnight and stored at −20 °C. Fixed cells were washed with phosphate-buffered saline (PBS) and stained in the dark with a solution containing a final concentration of propidium iodide (PI) (50 μg/mL) (Sigma), Triton X-100 (0.25%) and RNAse (1 mg/mL) for 1 hour at room temperature. DNA content was analyzed using a BD FACSCanto II flow cytometer (BD Biosciences) and data analysis was performed using FlowJo v.10 (TreeStar Inc).

Apoptosis was quantified as a percentage using an Annexin V-fluorescein isothiocyanate (FITC)/PI apoptosis detection kit (BD) according to the manufacturer’s instructions. Cells in the logarithmic growth phase were treated with the various drugs and combination and cells were harvested using Accutase (Sigma-Aldrich), and Annexin-V-FITC/PI labeling was performed according to the manufacturers’ instructions. The stained cells were analyzed using a BD FACSCanto II flow cytometer (BD Biosciences). The number of viable (annexin V−/PI−), apoptotic (annexin V+/PI−), and necrotic (annexin V+/PI+) cells were calculated with FlowJo v.10 (TreeStar Inc).

### Western blot analysis

Cells were plated in 6-well plates and treated the following day as indicated. Cells were lysed by incubation in RIPA lysis buffer supplemented with PMSF and protease inhibitors. Protein concentrations were normalized using a BCA protein assay (Pierce Biotechnology). Proteins were denatured using Laemmli denaturing buffer (Bio-Rad Laboratories) supplemented with β-mercaptoethanol and separated on a 12% polyacrylamide SDS-PAGE gel. Proteins were transferred to a PVDF membrane and immunoblotted using the antibodies described. Membranes were developed using Clarity™ Western ECL Substrate Biorad Chemiluminescence system (BioRAD #170-506). The following primary antibodies were used at a dilution of 1:1000: MIF antibody (Abcam #ab7207), CD74 antibody [LN2] (Abcam AB9514), p44/42 MAPK (Erk1/2) (L34F12) Mouse mAb (Cell Signaling #4696S), p-p44/42, AKT, Cleaved PARP (Asp214) Antibody (Cell Signaling), Anti-CXCR2 antibody (ABCAM #ab14935), Anti-Src antibody [EPR5496] (ABCAM #ab109381). In addition, the following primary antibodies were also used at a dilution of 1:2000: CD44 (anti CD44 Ab from Abcam #ab51037), p-AKT (Cell signaling #9271S), CXCR4 (Cell signaling). Secondary antibodies were purchased from BioRad Immun-Star Goat Anti-Rabbit (GAR)-HRP Conjugate (BioRad) or Polyclonal Goat Anti-Mouse Immunoglobulins HRP. Beta-Actin (Dako) was used as a housekeeping gene on each membrane to assess the integrity and quantity of the extracts.

### *In vivo* studies

Eight week old female Balb/c nude mice (*nu/nu*) were used for RN1 patient-derived xenograft model. Approximately 2 × 10^5^ RN1 cells were intracranially implanted into the mice brains (1.0 mm, 1.5 mm lateral from the bregma and 3.0 mm deep). Tumor burden was assessed by humanely euthanizing mice daily between 40–50 days. Tumor was embedded in paraffin, sectioned and stained with hematoxylin and eosin (H&E). Treatment commenced at 43 days after implantation. Mice (n = 8) were randomized to the treatment groups. Ibudilast was administered by oral gavage and was given daily throughout the duration of the experiment (until all control mice had died). TMZ was administered by intraperitoneal (i.p.) injection at a schedule of 5 days per week (2 weeks on, 2 weeks off) for the duration of the experiment (until all control mice had died)^37^. Mice were euthanized if the animals displayed neurological health indicators that met the institutional criteria for sacrifice. Animal experiments were approved by University of New South Wales Animal Care and Ethics Committee (16/86A) and conducted in accordance with the National Health and Medical Research Council Australian Code of Practice for the Care and Use of Animals for Scientific Purposes.

### Statistical analyses

One-way analysis of variance (ANOVA) with Tukey’s multiple comparison test was used to compare between treatment groups. Survival differences between groups were determined using Kaplan-Meier log rank analysis. Statistical analyses (not including proteomics analysis) were performed using Prism 7 (GraphPad). A two-tailed p < 0.05 was considered statistically significant. All data are expressed as mean ± SEM.

## Supplementary information

Dataset 1
