# Supplementary material for: Ibudilast sensitizes glioblastoma to temozolomide by targeting Macrophage Migration Inhibitory Factor (MIF)
Source: Sci Rep. 2019 Feb 27;9:2905. doi: 10.1038/s41598-019-39427-4 (PMC6393433; doi:10.1038/s41598-019-39427-4)
Supplement: Supplementary file 1 — Dataset 1 [file 41598_2019_39427_MOESM1_ESM.docx]

**Supplementary Figure 1**

**Ibudilast sensitizes glioblastoma to temozolomide by targeting Macrophage Migration Inhibitory Factor (MIF)**

**Running Title: Combined ibudilast and temozolomide enhance survival in GBM**

Wendy Ha^1^, Hatice Sevim-Nalkiran^2^, Ashraf M. Zaman^1^, Kazuko Matsuda^3^, Mustafa Khasraw^4^, Anna K. Nowak^5^, Liping Chung^6^, Robert C. Baxter^6^, Kerrie L. McDonald^1^

1. Cure Brain Cancer Foundation Biomarkers and Translational Research Group, Prince of Wales Clinical School, Lowy Cancer Research Centre, University of New South Wales, NSW, Australia

2. Department of Medical Biology, Faculty of Medicine, Recep Tayyip Erdogan University, Rize, Turkey

3. MediciNova Inc. La Jolla , CA, USA

4. Royal North Shore Hospital, Department of Medical Oncology, University of Sydney, St Leonards, NSW, Australia

5. School of Medicine, University of Western Australia, Crawley WA Australia

6. Kolling Institute of Medical Research, University of Sydney, St Leonards, NSW, Australia

**Corresponding Author:**

Kerrie L. McDonald

Cure Brain Cancer Foundation Biomarkers and Translational Research Group,

Level 2

Lowy Cancer Research Centre C25,

University of New South Wales,

NSW, Australia 2502

+61 2 9385 1471 (tel)

+61 293851510 (fax)

k.mcdonald@unsw.edu.au

**Figure Legend**

Original Western blot images shown in Fig. 4. Cells (BAH1) were treated with ibudilast, temozolomide or a combination of ibudilast and TMZ. Proteins were extracted after 8-days of treatment. (A) β-actin; (B) p42/44; (C) CD74; (D) p-AKT; (E)CXCR2; (F) AKT; (G) CD44 and p-P42/44; (H) cleaved PARP and (I) Src and MIF. All western blots experiments were repeated 3 times.


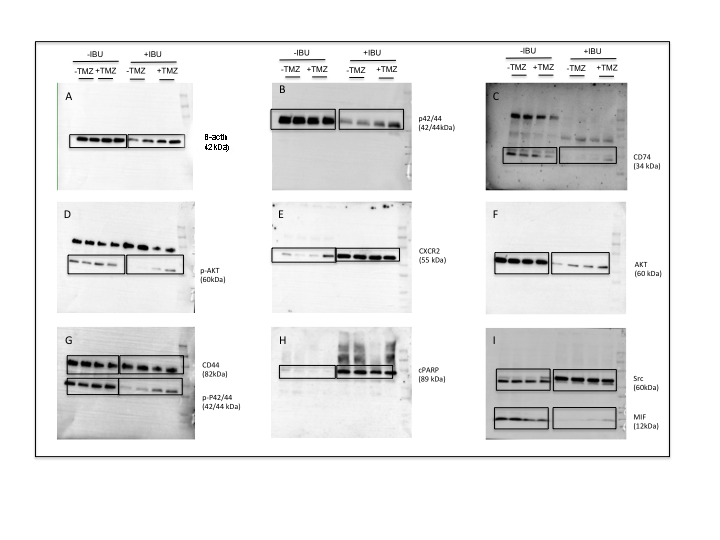


b-actin

(42 kDa)
